# Assessment of Protein Immunoexpression Associated with Tumor Proliferation and Invasion in Histological Subtypes of Unicystic and Conventional Ameloblastoma

**DOI:** 10.3390/ijms26031267

**Published:** 2025-01-31

**Authors:** Gabriela Cristina Avertano Rocha da Silveira, Rebeca Vieira Costa, Flavia Letícia Magalhães Lemos, Antonia Taiane Lopes de Moraes, Maria Sueli da Silva Kataoka, Vanessa Morais Freitas, Silvio Augusto Fernandes de Menezes, Ana Carolina Uchoa Vasconcelos, Adriana Etges, Fabricio Passador Santos, Vera Cavalcanti de Araújo, Sérgio de Melo Alves Júnior, Ruy Gastaldoni Jaeger, João de Jesus Viana Pinheiro

**Affiliations:** 1 Laboratory of Pathological Anatomy and Immunohistochemistry, School of Dentistry, Federal University of Pará, Belém 66075-110, PA, Brazil; gabriela.avertano.rocha@gmail.com (G.C.A.R.d.S.); rebecavcosta@outlook.com (R.V.C.); leticiamalemos@gmail.com (F.L.M.L.); sergioalves@ufpa.br (S.d.M.A.J.); 2 Oral Diagnosis Department, Piracicaba Dental School, University of Campinas (UNICAMP), Piracicaba 13414-903, SP, Brazil; taiane_moraes12@hotmail.com; 3 Cell Culture Laboratory, School of Dentistry, Federal University of Pará, Belém 66075-110, PA, Brazil; sukataoka@yahoo.com.br; 4 Institute of Biomedical Sciences, University of São Paulo, São Paulo 05508-000, SP, Brazil; vfreitas@usp.br (V.M.F.); rgjaeger@usp.br (R.G.J.); 5 Department of Periodontics, University Center of Pará, Belém 66060-232, PA, Brazil; menezes@cesupa.br; 6 Center for the Diagnosis of Diseases of the Mouth, School of Dentistry, Federal University of Pelotas, Pelotas 96010-610, RS, Brazil; carolinauv@gmail.com (A.C.U.V.); aetges@gmail.com (A.E.); 7 Department of Oral Pathology, São Leopoldo Mandic Institute and Research Center, Campinas 13045-755, SP, Brazil; fabricio.passadorsantos@slmandic.edu.br (F.P.S.); vcaraujo@usp.br (V.C.d.A.)

**Keywords:** ameloblastoma, immunohistochemistry, invadopodium, odontogenic tumor, pathology

## Abstract

The aim of this study was to verify whether the expression of proteins related to the formation of invadopodia, MT1-MMP, cortactin, Tks-4 and Tks-5 is associated with the degree of tumor invasiveness of different types of unicystic ameloblastomas. An immunohistochemical study was performed on 29 unicystic ameloblastoma (UA) samples, 9 conventional ameloblastoma (CAM) samples and 9 dental follicle (DF) samples. The potential for tumor invasiveness was assessed based on the immunoexpression of the following invadopodia-forming proteins: MT1-MMP, cortactin, Tks-4 and Tks5. Mural unicystic ameloblastoma (MUA) showed higher MT1-MMP, cortactin, Tks-4, and Tks-5 immunoexpression than luminal and intra-luminal types. Conventional ameloblastoma exhibited lower MT1-MMP, cortactin, and Tks-5 expression compared to MUA. MUA’s cystic capsule neoplastic cells had significantly higher MT1-MMP, cortactin, Tks-4, and Tks-5 expression than lumen cells. Dental follicles showed minimal expression. Neoplastic cells in the cystic capsule of mural unicystic ameloblastomas showed higher invadopodia-related protein expression than lumen and luminal/intraluminal cells, suggesting that proximity to the bone region influences the aggressive behavior of mural unicystic ameloblastomas more compared to other subtypes.

## 1. Introduction

Unicystic ameloblastoma is a variant of ameloblastoma characterized by a single cystic cavity, which may exhibit luminal proliferation [1]. Although benign, its mural variant demonstrates significant invasive potential, resembling conventional ameloblastoma according to the 2024 WHO classification [2]. Diagnosis requires clinical, radiographic, and comprehensive histopathological evaluation. Treatment is surgical, ranging from enucleation to resection, with greater attention to the mural variant due to its aggressiveness and long-term recurrence risk, even after 10 years [1].

Tumor invasion is directly associated with the ability of neoplastic cells to degrade and remodel the extracellular matrix (ECM), primarily through the action of matrix metalloproteinases (MMPs) [3,4]. Studies suggest that this process involves dynamic plasma membrane protrusions, known as invadopodia, which are rich in actin filaments [3,5,6,7]. Invadopodia formation is driven by epidermal growth factor receptor (EGFR) signaling and actin cytoskeleton-regulating proteins [8,9]. Essential molecules for invadopodia structure include actin nucleators, polymerization activators, cross-linking proteins, kinases, and scaffolding proteins, with Tks-4, Tks-5, cortactin, and MT1-MMP being particularly significant [6].

Scaffold proteins (from the Tks group) are present in various tissues and play diverse roles in both physiological and pathological processes. A prominent protein in this group is Tks-4, which is encoded by the SH3PXD2B gene [10,11]. Tks-4 was identified in the invadopodia of Src-transformed fibroblasts. When silenced, incomplete invadopodia formation occurred, with reduced actin polymerization and extracellular matrix degradation, suggesting its crucial role in invadopodia formation and function [11]. Tks-5, encoded by the SH3PXD2A gene, is also essential for invadopodia formation in cancer cells [12,13,14,15], interacting with proteins like Wiskott–Aldrich syndrome protein (N-WASP) and cortactin, which are involved in actin remodeling [5,16]. Cortactin, regulated by the CTTN gene, facilitates the formation of the actin network in invadopodia and plays a key role in the initiation, formation, maturation, and retraction phases of these structures [17]. Its elevated expression is common in cancer cells and is directly related to the processes of migration, invasion, and secretion of extracellular matrix proteases [18,19].

Studies show that the proteins Tks-4, Tks-5, cortactin, and MT1-MMP are closely related to the tumor invasion process [18,19,20,21,22,23]. Tks-4 and Tks-5 play a role in invadopodia formation, regulating the actin cytoskeleton through interactions with N-WASP and the Arp2/3 complex [18,19]. Cortactin, recruited to invadopodia, facilitates actin polymerization and contributes to cell migration and invasion, as well as promoting extracellular matrix degradation through interaction with MT1-MMP [17,18,20,21]. MT1-MMP, essential for matrix degradation, which activates soluble MMPs such as MMP-2, MMP-9, and MMP-13, enabling local and systemic matrix degradation during the invasive process [10,12,13,22,23].

Invadopodia formation plays a crucial role in tumor invasion, facilitating the degradation of the extracellular matrix and the dissemination of tumor cells. Previous studies have shown that proteins such as Tks-4, Tks-5, cortactin and MT1-MMP are directly involved in this process, and are often associated with tumors with invasive and aggressive behavior. However, the expression of these proteins in the different histological subtypes of unicystic ameloblastomas, which present distinct biological behaviors, has not yet been comprehensively investigated. Thus, this study aims to investigate the expression of Tks-4, Tks-5, cortactin and MT1-MMP proteins in the different histological subtypes of unicystic ameloblastomas and correlate it with the specific biological behavior of each subtype.

## 2. Results

The distribution of samples from the different types of ameloblastoma into groups was performed after the collection of clinical and anatomopathological data, which can be seen in Table 1.

All samples from the conventional ameloblastoma (CAM), mural unicystic ameloblastoma (MUA), luminal unicystic ameloblastoma (LUA), intraluminal unicystic ameloblastoma (IUA), and dental follicle (DF) groups showed expression of the proteins MT1-MMP, cortactin, Tks-4, and Tks-5 in neoplastic parenchymal cells as well as in the epithelium of the DF. The immunohistochemical staining of the studied proteins was located in the cords and islands of the epithelium of odontogenic tumors and the dental follicle.

### 2.1. Immunoexpression of MT1-MMP

**Figure 1:**
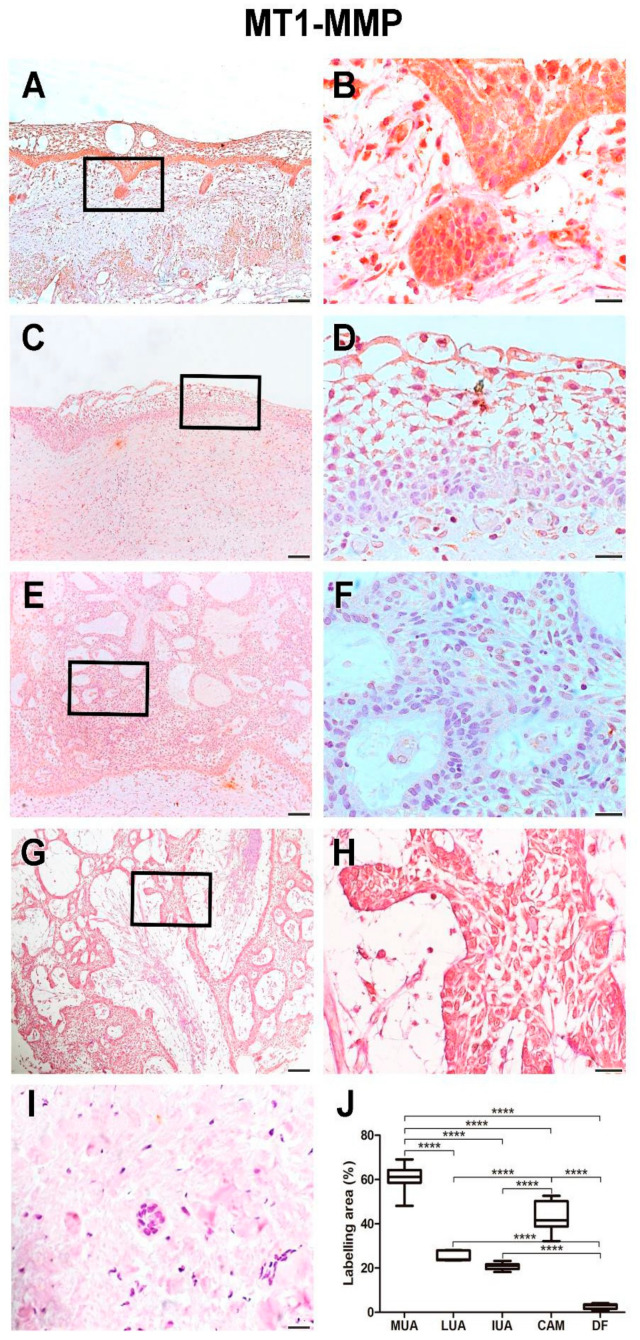
MT1-MMP expression in MUA (**A**,**B**), LUA (**C**,**D**), IUA (**E**,**F**), CAM (**G**,**H**) and DF (**I**) groups. Magnification: 100 and 630×. Scale bars: 20 and 100 µm. Comparison of MT1-MMP immunoexpression between the samples of MUA, LUA, IUA, CAM and DF, **** *p* < 0.0001 (**J**).

**Figure 2:**
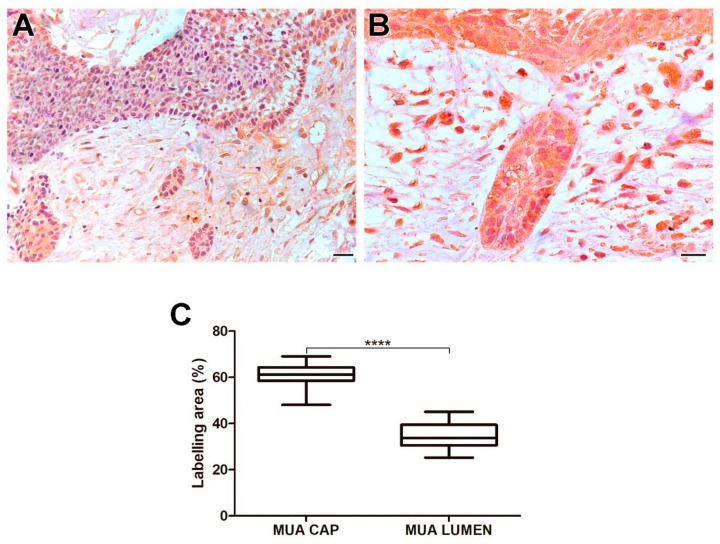
MT1-MMP expression in neoplastic MUA cells in the cystic capsule region and neoplastic MUA cells in the lumen region (**A**,**B**). Scale bars: 20 and 100 µm. Comparison of MT1-MMP immunoexpression between the capsule and lumen areas of MUA, **** *p* < 0.0001 (**C**).

The MUA group exhibited a higher immunoexpression rate of MT1-MMP compared to the LUA (*p* < 0.0001), IUA (*p* < 0.0001), and CAM (*p* < 0.0001) groups. Neoplastic cells in the cystic capsule of MUA showed a greater percentage of MT1-MMP expression compared to the lumen (*p* < 0.0001). MT1-MMP expression was observed in the cytoplasm of tumor parenchyma cells. Histological images and analysis of MT1-MMP comparisons between the UA, CAM, and DF groups are presented in Figure 1, while the comparison of immunomarking rates between the neoplastic cell regions in the cystic capsule and lumen of MUA can be seen in Figure 2.

### 2.2. Immunoexpression of Cortactin

**Figure 3:**
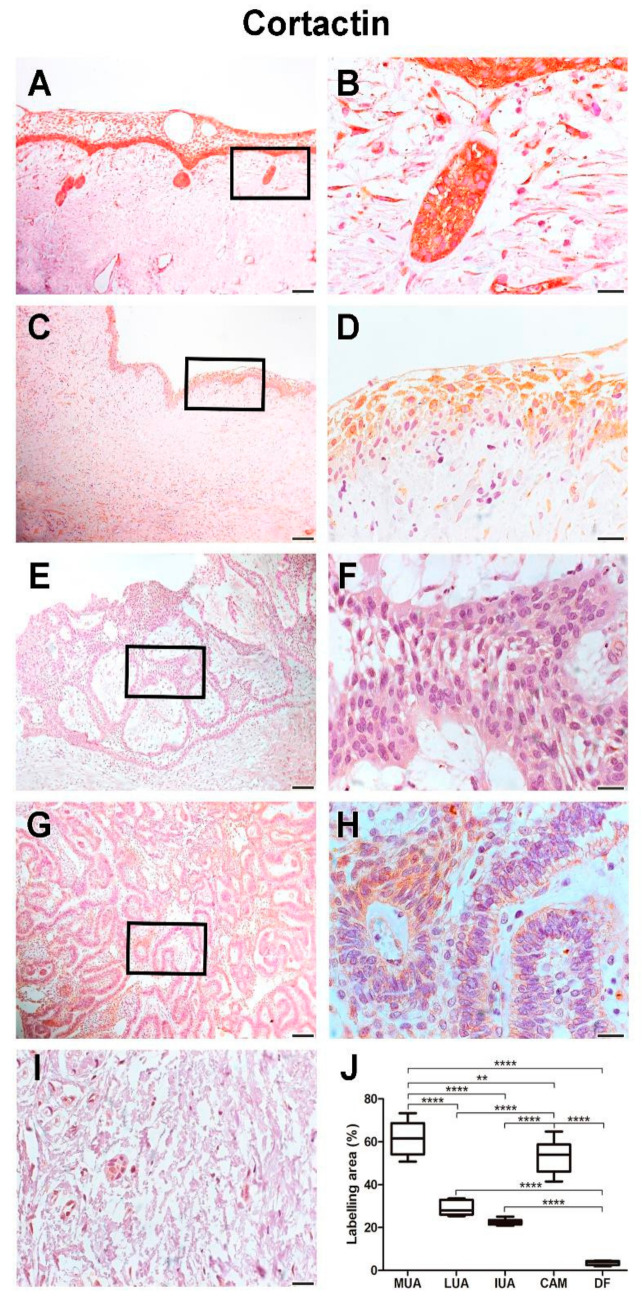
Cortactin expression in MUA (**A**,**B**), LUA (**C**,**D**), IUA (**E**,**F**), CAM (**G**,**H**) and DF (**I**) groups. Magnification: 100 and 630×. Scale bars: 20 and 100 µm. Comparison ofcortactin immunoexpression between the samples of MUA, LUA, IUA, CAM and DF, ** *p* < 0.01, **** *p* < 0.0001 (**J**).

**Figure 4:**
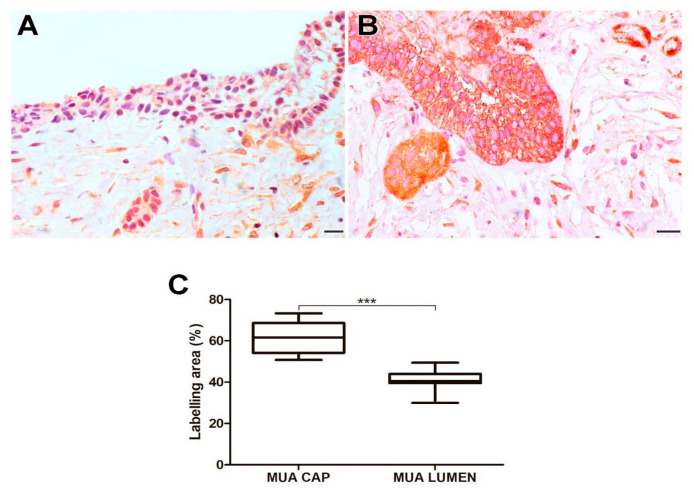
Cortactin expression in neoplastic MUA cells in the cystic capsule region and neoplastic MUA cells in the lumen region (**A**,**B**). Scale bars: 20 and 100 µm. Comparison of MT1-MMP immunoexpression between the capsule and lumen areas of MUA, *** *p* < 0.001 (**C**).

MUA exhibited a higher immunoexpression rate of cortactin compared to LUA (*p* < 0.0001), IUA (*p* < 0.0001), and CAM (*p* < 0.01). Neoplastic cells in the cystic capsule of MUA demonstrated a higher percentage of cortactin expression compared to the lumen (*p* < 0.001). Cortactin marking was identified in the cytoplasm of tumor parenchyma cells. Histological images and analysis of cortactin comparisons between the UA, CAM, and DF groups are presented in Figure 3, while the comparison of immunomarking rates between the neoplastic cell regions in the cystic capsule and lumen of MUA can be seen in Figure 4.

### 2.3. Immunoexpression of Tks-4

**Figure 5:**
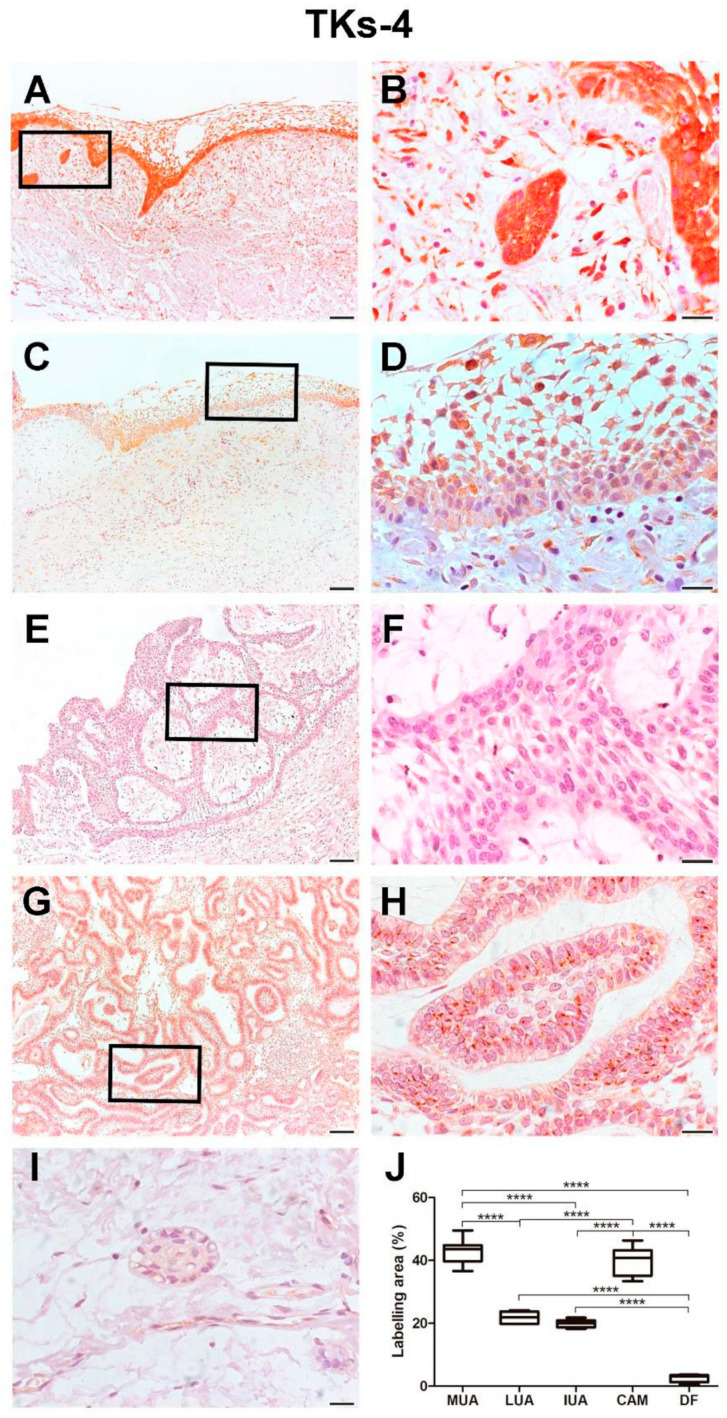
Tks-4 expression in MUA (**A**,**B**), LUA (**C**,**D**), IUA (**E**,**F**), CAM (**G**,**H**) and DF (**I**) groups. Magnification: 100 and 630×. Scale bars: 20 and 100 µm. Comparison ofcortactin immunoexpression between the samples of MUA, LUA, IUA, CAM and DF, **** *p* < 0.0001 (**J**).

**Figure 6:**
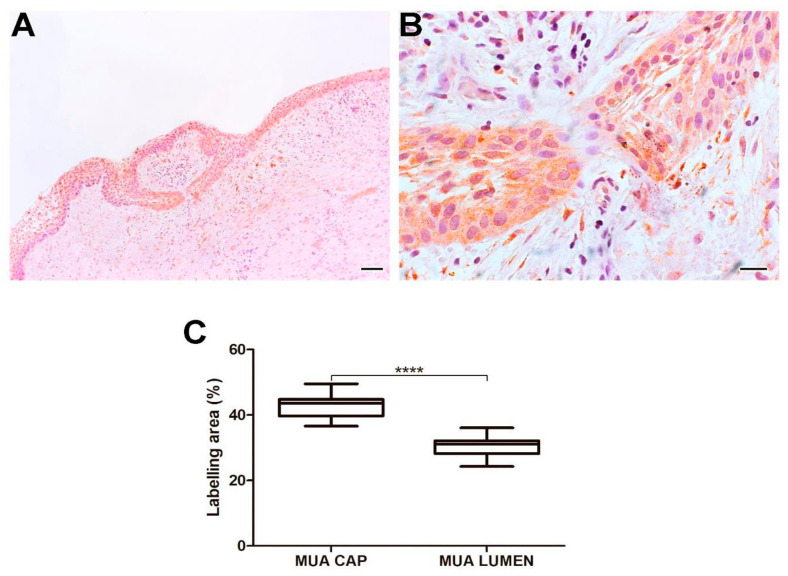
Tks-4 expression in neoplastic MUA cells in the cystic capsule region and neoplastic MUA cells in the lumen region (**A**,**B**). Scale bars: 20 and 100 µm. Comparison of MT1-MMP immunoexpression between the capsule and lumen areas of MUA, **** *p* < 0.0001 (**C**).

MUA displayed a higher immunoexpression rate of Tks-4 compared to LUA (*p* < 0.0001) and IUA (*p* < 0.0001). No statistical difference was found when comparing Tks-4 immunoexpression in the neoplastic cells of the cystic capsule of MUA with the cells of the CAM group. Neoplastic cells in the cystic capsule of MUA showed a greater percentage of Tks-4 expression compared to the lumen (*p* < 0.0001). Tks-4 expression was observed in the cytoplasm of tumor parenchyma cells. Histological images and analysis of Tks-4 comparisons between the UA, CAM, and DF groups are presented in Figure 5, while the comparison of immunomarking rates between the neoplastic cell regions in the cystic capsule and lumen of MUA can be viewed in Figure 6.

### 2.4. Immunoexpression of Tks-5

**Figure 7:**
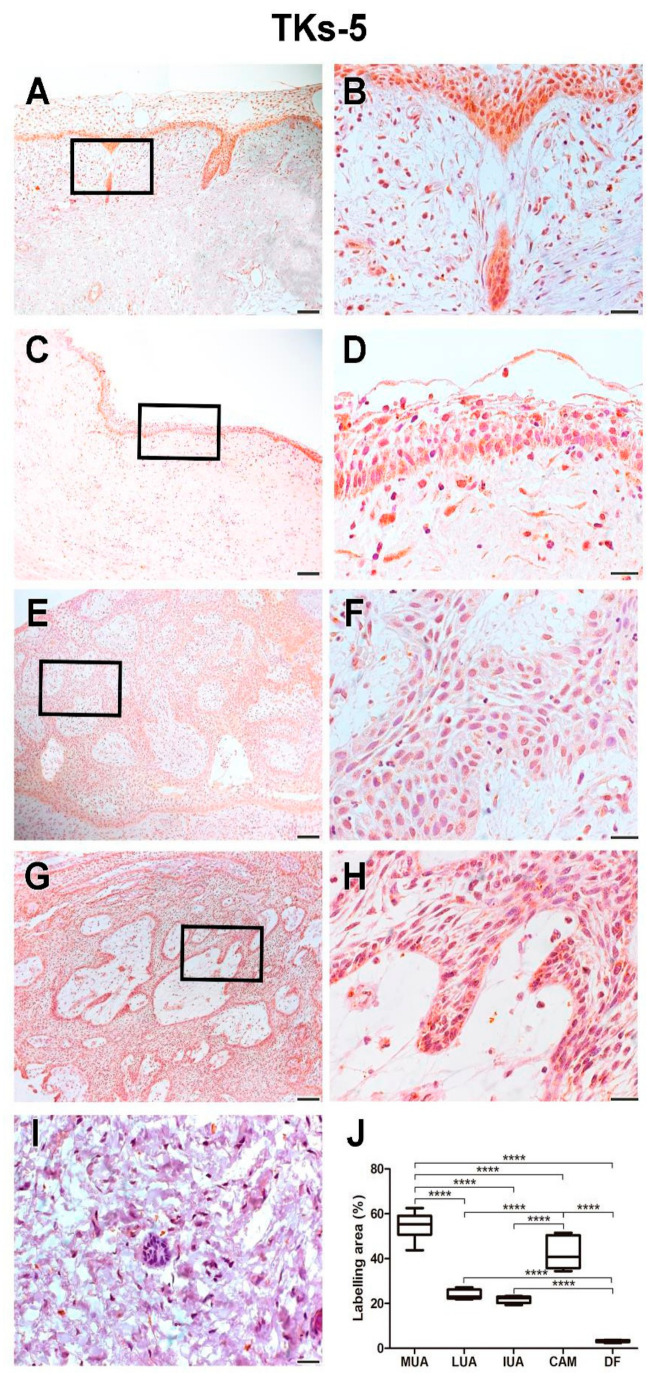
Tks-5 expression in MUA (**A**,**B**), LUA (**C**,**D**), IUA (**E**,**F**), CAM (**G**,**H**) and DF (**I**) groups. Magnification: 100 and 630×. Scale bars: 20 and 100 µm. Comparison ofcortactin immunoexpression between the samples of MUA, LUA, IUA, CAM and DF, **** *p* < 0.0001 (**J**).

**Figure 8:**
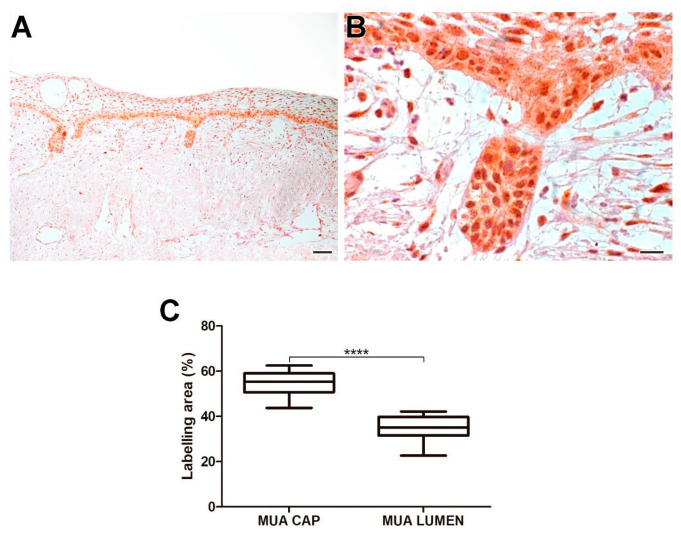
Tks-5 expression in neoplastic MUA cells in the cystic capsule region and neoplastic MUA cells in the lumen region (**A**,**B**). Scale bars: 20 and 100 µm. Comparison of MT1-MMP immunoexpression between the capsule and lumen areas of MUA, **** *p* < 0.0001 (**C**).

MUA had a higher immunoexpression rate of Tks-5 compared to LUA (*p* < 0.0001), IUA (*p* < 0.0001), and CAM (*p* < 0.0001). Neoplastic cells in the cystic capsule of MUA demonstrated a higher percentage of Tks-5 expression compared to the lumen (*p* < 0.0001). Tks-5 expression was identified in the cytoplasm of tumor parenchyma cells. Histological images and analysis of Tks-5 comparisons between the UA, CAM, and DF groups are presented in Figure 7, while the comparison of immunomarking rates between the neoplastic cell regions in the cystic capsule and lumen of MUA can be seen in Figure 8.

## 3. Discussion

The clinical data for the studied UA samples showed a higher prevalence of the mural subtype, with most patients being in their third decade of life, the majority of whom were also female. The most prevalent location was the mandible. In the CAM samples, most patients were in their fourth decade of life, and the predominant location was also the mandible.

Some studies [24,25] observed a higher prevalence of UA in young patients, with an average age in the third decade of life, and a higher incidence in the posterior region of the mandible, with most cases being classified as the mural variant. Another study [26] found that CAM is more common in patients between the third and seventh decades of life, with the mandible being the most affected site. The clinical data from the present study support the findings in the literature. However, according to the 2024 WHO classification, approximately 50% of UA cases are diagnosed in the second decade of life, with a slight predominance of these tumors in males [2].

Focal degradation, a phenomenon associated with cellular invasiveness, is mediated by invadopodia, finger-like protrusions enriched with actin filaments, and is linked to the initiation of invasion and activation of MMPs [7,27].

The formation of invadopodia is characterized by the localization of cortactin on the cell’s ventral surface, recruitment of MT1-MMP, and the presence of degraded foci in the underlying matrix. This mechanism is assisted by the scaffold proteins Tks-4 and Tks-5 [7,27].

In this study, the hypothesis was raised that MUA has higher expression of invadopodia-forming proteins, MT1-MMP, cortactin, Tks-4, and Tks-5, when compared to other subtypes of unicystic ameloblastoma, due to the location of neoplastic cells being present in the cystic capsule.

Using immunohistochemistry, it was found that the samples of MUA, LUA, IUA, CAM, and DF expressed the proteins MT1-MMP, cortactin, Tks-4, and Tks-5. Furthermore, high immunoexpression was observed in MUA, LUA, IUA, and CAM when compared to the control group, DF. This was expected, as these proteins are associated with neoplastic invasion, which is not the case for DF.

In turn, MUA exhibited higher expression of MT1-MMP, cortactin, and Tks-5 compared to all the groups studied, with the expression of Tks-4 being the only one not higher in comparison to CAM.

These results possibly indicate the involvement of these proteins in the local invasiveness of MUA. Our main focus was to relate the studied proteins to invadopodium formation, as there is strong evidence in the literature linking the formation and activity of these structures to the pathogenesis of both benign and malignant tumors [21,28].

In our findings, there was a superexpression of MT1-MMP, with predominant cytoplasmic labeling, membrane localization, and slight nuclear labeling in the tumor epithelium of MUA. It is known that MT1-MMP is associated with the invasive process of certain benign and malignant neoplasms, and it is required to enhance invadopodium formation, thereby promoting cellular invasion [21,28,29].

MT1-MMP is a membrane-bound MMP associated with localized degradation of the extracellular matrix and is classically known for activating other MMPs, such as MMP-2 and MMP-9 [29,30]. One of the factors responsible for stabilizing the intracellular response triggered by MT1-MMP are the TKs-4 and 5 molecules [29,31].

Among the Tks, Tks-4 has been classically associated with the recruitment of MT1-MMP to the cell membrane, indirectly participating in the lysis activity of invadopodia [13]. In our analysis, we observed elevated immunoexpression of Tks-4 in MUA, with intense cytoplasmic labeling in the tumor epithelial cells located in the cystic capsule. These findings were similar in CAM. The high expression of MT1-MMP and Tks-4 in MUA could be associated with its local invasiveness and higher recurrence rate reported in the literature [2].

In relation to invadopodia, two other proteins are important for the digitiform projection of the cell membrane into the extracellular matrix: cortactin and Tks-5. In this regard, invadopodia are specifically identified by microscopy as invasive proteolytic protrusions containing Tks-5 and cortactin [32].

Our results revealed high expression of cortactin in the MUA samples, with intense cytoplasmic immunostaining, particularly in the neoplastic epithelial cells of the cystic capsule. As mentioned, the expression and recruitment of cortactin are crucial for the initial events in invadopodia formation, regulating the polymerization of F-actin filaments [13]. Additionally, regarding cytoskeletal modifications, we observed high immunoexpression of Tks-5 in the MUA, especially in the neoplastic cells located in the cystic capsule. It seems important to emphasize that the high expression of cortactin and Tks-5 likely collaborate with each other for potential invadopodia formation [33].

In summary, we suggest that the proteins MT1-MMP, cortactin, Tks-4, and Tks-5 may be directly related to the local invasiveness mechanism of MUA. The expression of these proteins in MUA increases the potential for invadopodia formation, thereby enhancing tumor invasiveness.

Another important point to highlight is that, as suggested by Pinheiro et al., 2004 [34], and, in the first chapter of Vieira et al., 2024, [35]’s thesis, the proximity of neoplastic cells to the resorbing bone surrounding the lesion would result in the release of growth factors and cytokines that would stimulate the expression of the studied molecules. This biological phenomenon would explain the higher expression of some proteins in MUA compared to CAM and other UA subtypes [36,37,38].

The biological mechanisms underlying invadopodia formation are highly dependent on the coordinated interaction between key proteins, such as cortactin, Tks-4, Tks-5, and MT1-MMP. These mechanisms involve cortactin-mediated F-actin polymerization, which organizes the cytoskeletal architecture required for the formation of invasive protrusions. Tks-4 and Tks-5 act as scaffold proteins that stabilize protein complexes and recruit MT1-MMP to the cell membrane, facilitating localized degradation of the extracellular matrix. Furthermore, in the tumor microenvironment, growth factors and cytokines released by the interaction between neoplastic cells and resorbed bone tissue can stimulate the expression of these proteins, enhancing invasive activity [13,29,33].

This study is preliminary in nature, and the limitations observed, such as the small sample size in some subgroups and the observational nature, indicate the need for further investigation. Future studies should include functional analyses to explore in more depth the causal relationships between the overexpression of the investigated proteins and the local invasiveness of the tumors. Another limitation is the relatively small sample size in some subgroups, which may hinder the generalization of the findings and limit the ability to detect statistical differences in comparative analyses. Additionally, while associations were observed between the expression of certain proteins and tumor invasiveness, the observational nature of the study prevents the establishment of causal relationships. Therefore, caution is needed when extrapolating these findings to clinical practice.

## 4. Materials and Methods

### 4.1. Ethical Aspects

This study was conducted in accordance with the criteria established by the Research Ethics Committee for Human Beings at the Institute of Health Sciences, Federal University of Pará—ICS/UFPA, and was approved under protocol number 4.570.860, in compliance with the Declaration of Helsinki [39].

### 4.2. Study Sample

Immunohistochemical reactions were performed on 47 samples derived from humans: 29 samples of UA, 9 samples of CAM, and 9 samples of DF. The samples and clinical data on the patients were collected from the archives of the Center for Diagnosis of Oral Diseases (CDDB) at the School of Dentistry of the Federal University of Pelotas, the São Leopoldo Mandic Research Center and Institute, and the Laboratory of Pathological Anatomy and Immunohistochemistry at the School of Dentistry of the Federal University of Pará. All samples were diagnosed based on image analysis and trans-surgical exams, combined with the histological analysis of the entire lesion, to rule out epithelial tumor invasions into the cystic capsule in cases of MUA. The total sample was divided into five groups according to the WHO classification (2024) for head and neck tumors as follows: the MUA group, with 14 samples of the mural subtype; the LUA group, with 7 samples of the luminal subtype; and the IUA group, with 8 samples of the intraluminal subtype. The CAM group included 9 samples microscopically diagnosed as conventional ameloblastoma, which served as a positive control due to the established expression of the proteins MT1-MMP, cortactin, Tks-4, and Tks-5 in this tumor [21]. Additionally, the DF group was included, consisting of 9 samples of dental follicles, which are normal dental tissues without cystic or neoplastic alterations [2]. Following the same diagnostic standard, all cases of UA and CAM were diagnosed based on clinical, radiographic, surgical, and histopathological aspects.

### 4.3. Immunohistochemistry

For immunohistochemical analysis, the histological slides were deparaffinized in xylene and hydrated in decreasing concentrations of ethanol. The samples were then immersed in a solution of 3% hydrogen peroxide and methanol (1:1) to block endogenous peroxidase activity. Antigen retrieval was performed with citrate buffer (pH 6.0) in a Pascal pressure chamber (Dako Cytomation, Carpinteria, CA, USA) for 30 s at 125 °C. After treatment with 1% bovine serum albumin (Sigma-Aldrich, St. Louis, MO, USA) in phosphate-buffered saline for 40 min, the sections were incubated for 1 h in a humid chamber at room temperature with primary antibodies: Anti-Tks-5 (1:50 Sigma^®^), Anti-Tks-4 (1:50 Sigma^®^), Anti-cortactin (1:600 Abcam^®^, Cambridge, UK), and Anti-MT1-MMP (1:300 R&D Systems^®^, Minneapolis, MN, USA). The slides were then incubated and treated at room temperature with a dextran polymer-based complex (Reveal, Spring Bioscience, Pleasanton, CA, USA), and diaminobenzidine (DAB) was used as the chromogenic agent (Liquid DAB + Substrate, Spring Bioscience, Pleasanton, CA, USA). Finally, the slides were counterstained with Mayer’s hematoxylin (Sigma-Aldrich) and mounted with Permount mounting medium (Fisher Scientific, Fair Lawn, NJ, USA).

### 4.4. Evaluation of Immunostaining

Five bright-field images were acquired from each sample using an AxioScope microscope (Carl Zeiss, Oberkochen, Germany), equipped with a color CCD camera AxiocCam HRC (Carl Zeiss**^®^**), randomly. The obtained images were captured at the same magnification (400×) and saved in TIFF format. In the case of MUA, 10 images were acquired from each sample: 5 images from cells that had invaded the cystic capsule and 5 from cells present in the lumen. The areas stained by diaminobenzidine were analyzed using the “Immunohistochemistry (IHC) Image Analysis Toolbox” (Jie Shu, Guoping Qiu, and Mohammad Ilyas, https://imagej.net/ij/plugins/ihc-toolbox/index.html (accessed on 10 January 2023)) of the ImageJ software (public domain software, version 14), developed by Wayne Rasband (NIMH, NIH, Bethesda, MD, USA, https://imagej.net/ij/ (accessed on 10 January 2023)). The evaluation of immunostaining was performed by measuring the fraction (%) of immunostaining in neoplastic cells with DAB relative to the total quantified parenchyma for the antibodies MT1-MMP, cortactin, Tks-4, and Tks-5. The analysis of the average percentage of staining in the five fields from the samples was conducted using GraphPad Prism 5 software (GraphPad Software Inc., San Diego, CA, USA, accessed on 20 March 2023).

### 4.5. Statistical Analysis

The Shapiro–Wilk test was used to assess the normality of the data distribution. For parametric data, analysis of variance (ANOVA) was performed, followed by Bonferroni correction. The difference between the areas of the MUA capsule and the lumen was evaluated using the Mann–Whitney test. A 95% confidence interval was assumed (*p* = 0.05).

## 5. Conclusions

Neoplastic cells in the cystic capsule of MUAs exhibited higher expression of proteins associated with invadopodia formation (MT1-MMP, cortactin, Tks-4, and Tks-5) compared to cells in the lumen of MUAs and those from other subtypes (LUA, IUA, and CAM), except for Tks-4, which was highly expressed in both MUA and CAM. These findings suggest that cells closer to the bone region may be influenced by molecules released during bone resorption, leading to increased expression of these proteins and contributing to a more aggressive biological behavior in MUA compared to other UA subtypes.

## Figures and Tables

**Table 1:** Clinical and demographic characteristics of ameloblastoma samples: histological subtype, sex, age, and anatomical location of the lesion.

	Unicystic Ameloblastoma			Conventional Ameloblastoma	Dental Follicle
	Mural	Luminal	Intraluminal		
Sex (n = 47)					
Female	8	5	3	4	4
Male	3	2	4	5	5
NR*	3	0	1	0	0
Age (n = 47)					
00–09	1	0	2	0	0
10–19	1	3	4	0	5
20–29	7	3	1	1	2
30–39	0	0	0	4	1
40–49	0	0	0	2	0
50–59	0	1	0	2	0
60–69	0	0	0	0	0
70–79	0	0	0	0	0
80–89	0	0	0	0	0
NR*	4	0	1	0	1
Anatomical Location (n = 47)					
Maxilla	1	0	0	0	0
Mandible	9	5	5	10	8
NR*	5	1	1	0	2

NR*: not related.

## Data Availability

Data supporting the findings of this study is available from the corresponding author upon reasonable request.

## Abbreviations

The following abbreviations are used in this manuscript:

CAM	Conventional Ameloblastoma
WHO	World Health Organization
UA	Unicystic Ameloblastoma
MUA	Mural Unicystic Ameloblastoma
LUA	Luminal Unicystic Ameloblastoma
IUA	Intraluminal Unicystic Ameloblastoma
DF	Dental Follicle
MMPs	Matrix Metalloproteinases
EGFR	Epidermal Growth Factor Receptor
ECM	Extracellular Matrix
